# Letter to the editor: Evidence on school closure and children’s social contact: useful for coronavirus disease (COVID-19)?

**DOI:** 10.2807/1560-7917.ES.2020.25.17.2000758

**Published:** 2020-04-30

**Authors:** Michele Poletti, Andrea Raballo

**Affiliations:** 1 Department of Mental Health and Pathological Addiction, AUSL-IRCCS of Reggio Emilia, Reggio Emilia, Italy; 2 Division of Psychiatry, Clinical Psychology and Rehabilitation, Department of Medicine, University of Perugia, Perugia, Italy; 3 Center for Translational, Phenomenological and Developmental Psychopathology, Perugia University Hospital, Perugia, Italy

**Keywords:** Covid-19, school closure, quarantine, social contact, social isolation, mental health

**To the editor:** We read with interest the recent rapid evidence review by Brooks and colleagues about the impact of unplanned school closure on children’s social contact [1]. This review substantially aimed at investigating if children adhere to social isolation or continue to mix with others, limiting the effects of school closure and of quarantine. This is an important topic, the of dark side of which resides on effects of prolonged school closure on well-being of children, poorly considered in the current public debate on management of coronavirus disease (COVID-19) [2]. In this perspective, mental health of children and adolescents undergoes a sudden stress test during school closure, with increased risk of loneliness, addiction to videogames and binge watching, alteration of circadian rhythms, direct or assisted domestic violence, and academic achievement gaps. Especially for the latter, inequalities related to socioeconomic status and differences related to pre-existing vulnerabilities will be further amplified [2-4].

Brooks and colleagues [1] clearly reviewed literature on unplanned school closure to extract potential clues in relation to the management of the current COVID-19 pandemic, but generalisability of findings for this aim is questionable, as acknowledged by authors in the discussion. Two intertwined main obstacles for the generalisability of findings relate to the geographic expansion of the pandemic and to the temporal duration of school closures. Examined studies were almost entirely based on experiences in context with the 2009 influenza A(H1N1) pandemic or other influenza-like outbreaks, that did not have the scale of the COVID-19 pandemic, being geographically limited in some areas. Moreover, duration of previous experiences of school closures ranged from 1 day to 2 weeks, while the current COVID-19 pandemic is causing much longer school closures. For example, in Italy, children and adolescents will be away from schools for almost 6 months (3 months of school closure plus 3 months of usual summer vacation), with the vague possibility of summer camps, in our opinion actually with low probability to be allowed and implemented.

Therefore, findings based on geographically and temporally limited school closures may be poorly informative for a pandemic at the scale of COVID-19 and consequent temporally extended school closures. This is the case because risk perception of children and also of their parents (other factors that Brooks and colleagues found related to the respect of social isolation [1]) is clearly different in the two scenarios and therefore also adherence to social isolation is probably different [5]. In this perspective, one of the main conclusions of the review, i.e. during school closure children continue having social contacts with others, is very poorly informative to predict adherence to social isolation of children during school closure along the COVID-19 pandemic.

Instead, considering how much social isolation may affect mental health of children and adolescents [2-4], we strongly suggest that medical, educational and economical authorities should implement as soon as possible strategic plans for a progressive re-start of school or educational activities. This re-start should ensure a calculated trade-off between risk of COVID-19 infection and reduction of risk for children’s well-being, especially for more vulnerable subgroups, as those of families with low socioeconomic status and those with pre-existing mental health problems or learning difficulties.
